# The difference of disease perception by juvenile idiopathic arthritis patients and their parents: analysis of the JAMAR questionnaire

**DOI:** 10.1186/s12969-015-0063-3

**Published:** 2016-01-06

**Authors:** Federica Vanoni, Joan-Carles Suris, Annette von Scheven-Gête, Béatrice Fonjallaz, Michaël Hofer

**Affiliations:** Unité Romande d’Immuno-Rhumatologie Pédiatrique (URIRP), Département Médico - Chirurgical de Pédiatrie (DMCP), Centre Hospitalier Universitaire Vaudois CHUV, University of Lausanne, Rue du Bugnon 46, 1011 Lausanne, University of Geneva, Geneva, Switzerland; Groupe de recherche sur la santé des adolescents (GRSA), Institut universitaire de médecine sociale et préventive (IUMSP), Bâtiment Biopôle 2, Rte de la Corniche 10, 1010 Lausanne, Switzerland; Ligue genevoise contre le rhumatisme, Rue Merle d’Aubigné 22, 1207 Genève, Switzerland

**Keywords:** JAMAR, JIA, Perception, Disagreement

## Abstract

**Background:**

The JAMAR (Juvenile Arthritis Multidimensional Assessment Report) has been developed to evaluate the perception of the patient and his parents on different items: well-being, pain, functional status, quality of life, disease activity, disease course, side effects of medication, therapeutic compliance and satisfaction with illness outcome.

Our aim was to compare disease’s perception by JIA patients and their parents.

**Methods:**

We included into the study 100 consecutive patients over 7 years of age. We asked both parent and child to complete the JAMAR questionnaire. For each patient we recorded demographic and disease related data. We examined the level of disagreement between children and parents for the quantitative items of the JAMAR: VAS Pain, VAS Disease Activity, VAS Well Being, Juvenile Arthritis Functional Score, HRQoL. Then we looked for a relation between discordance-rate and demographic and clinical variables.

**Results:**

Children and parents’ median scores for all five items were similar. Individual dyads agreement was low, with a large amount of pairs (80) discordant for at least one item.

We found higher MD VAS and JADAS in more discordant dyads, suggesting that when the disease is more active discordance between child and parent increase.

**Conclusion:**

The JAMAR questionnaire is an important tool that helps clinicians to detect divergent child and parent’s disease perceptions. It is essential that both patients and parents fill the JAMAR questionnaire for a complete clinical and psychosocial evaluation.

**Electronic supplementary material:**

The online version of this article (doi:10.1186/s12969-015-0063-3) contains supplementary material, which is available to authorized users.

## Background

In recent years there has been an increasing interest in parent/patient-centred reported outcomes in juvenile idiopathic arthritis (JIA)[1–3]. These are important measures in patient assessment because they reflect the parents and children’s perception of the disease course and the effectiveness of medical interventions, and may contribute significantly to medical decisions and to improving patient care.

The Juvenile Arthritis Multidimensional Assessment Report (JAMAR) evaluates patient and parents’ disease perception. JAMAR is proposed for use as only proxy-report for patients younger than 7 years and as both self and proxy report for patients aged 7–18 years [4].

The literature shows conflicting results on disagreement between children with JIA and their parents in rating disability, pain, well-being and Health Related Quality of Life (HRQoL) [5–8].

In clinical practice, relying only on parent-reports gives us only partial information on the disease. It is therefore of great importance to encourage children’s ability to self-report verbally and in writing within paediatric care [9]. The parents’ perception of their child’s disease and their estimation of its impact on daily life affects health care utilization, so it is pivotal to increase the knowledge about the relationship between parent and child reporting. Parental perception can also influence the adolescent use of health care services and parents are expected gradually to relinquish their care giving responsibilities to their child. A different perception of the disease may also reflect family suffering, and a lack of communication between the patient and his/her parents. Therefore gaining more insight into child-parent disagreement is important in patient care.

The aim of this study was to examine the level of disagreement between children and parents for the quantitative items of the JAMAR: VAS Pain (VAS P), VAS Disease Activity (VAS DA), VAS Well Being (VAS WB), Juvenile Arthritis Functional Score (JAFS), HRQoL. A further aim was to identify factors associated to discordance.

## Methods

Each consecutive JIA patient over 7 or 8 years of age (depending on the child’s capacity to fill in the questionnaire on his/her own) seen at the paediatric rheumatology units of Lausanne, Geneva, Neuchâtel and Sion from May 2012 to June 2013 was included into the study. We asked both the accompanying parent and the child to independently complete a French parent or child-version of the JAMAR. As some patients came to our clinic more than once during the study period, we considered only the first filled in questionnaire.

For each patient we recorded demographic and disease related data (International League Against Rheumatism (ILAR) category, disease activity, number of active joints, erythrocyte sedimentation rate (ESR), physician centred measures) and calculated the Juvenile Arthritis Disease Activity Score (JADAS-71) [10]. The JADAS combines two physician-centred measures (physician global assessment and active joint count), one parent/patient-centred measure (parent/patient global assessment), and ESR. We calculated the median score for the VAS P, VAS DA, VAS WB, JAFS, HRQoL for children’s and parents’ groups.

We calculated the difference between child and parent scores. We defined as discordant the pairs that had a difference greater or equal to one for the VAS [5] and a discordance in at least one answer for the JAFS and HRQoL, leading to a difference of one or more in these scores. We divided our population in six groups depending on the number of discordant items: in group A pairs were concordant for all items, in group B pairs were discordant for one item, in group C for two items, in group D for three items, in group E for four items, and in group F for all items.

We compared demographic (age at disease’s onset, age at visit, disease duration) and clinical variables (number of active joints, physician evaluation of disease activity (MD VAS), JADAS) between these six groups.

The Ethics Committee approved this study and we obtained written informed consent from parents and patients.

### Statistical analysis

We calculated children and parents’ median scores for the five items.

Comparisons among the six groups for each variable were performed using one-way ANOVA and Tukey’s post hoc test. Statistical significance was set at *p* < 0.05.

## Results

A total of 100 child/parent pairs were included in the study. Median age at visit was 13.3 years, median age at disease onset was 7.9 years, median disease duration was 4.7 years. There was a predominance of females (72 females, 28 males) with a sex ratio of 2.6. Statistical analysis of demographic variables showed no significant differences between groups for age at visit/at diagnosis, duration of disease and number of active joints (Additional file 1 Figure S1). Median number of actives joints was 0 (range 0–11), median MD Global VAS was 1 (range 0–8) and median JADAS was 4 (range 0–18). All the subtypes of JIA were represented with predominance for oligoarticular (39 %) and enthesitis related JIA (36 %).

First, we analysed discrepancy between children and parents’ median scores of all five items (VAS P, VAS DA, VAS WB, JAFS, HRQoL): the differences between them were not statistically significant.

Then we looked individually at each patient-parent dyads. There were differences between child and parent’s scores for at least one item in 80 pairs (80 %). For the five items the differences were both in favour of children and parents depending of the duo. There was an increase in age at disease onset, age at visit, disease duration, number of active joints, MD VAS and JADAS with the increasing number of discordant items (Additional file 2 Table S1).

**Fig. 1:**
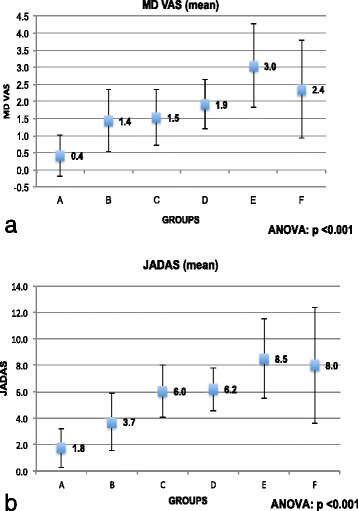
**a** MDVAS and **b** JADAS-71 in the six groups with results of ANOVA test between groups

Analysis of MD VAS and JADAS showed significant differences between groups (Fig. 1), but no differences were found for the number of active joints (Additional file 1 Figure S1). Post hoc analysis showed that MD VAS was significantly higher in group E than in group A (Additional file 3 Table S2); JADAS was significantly higher in groups D, E and F than in group A and in group E than in groups A and B (Additional file 4 Table S3).

## Discussion

We found substantial agreement between children’s and parents medians scores in all evaluated items, as already described [4]. However, individual dyads duo agreement was low, with a large amount of pairs (80) discordant for at least one item. Discordance in such areas as functional ability, pain and overall well-being suggests that parents and children may have a different perception of the disease.

Our results show an increased number of items with disagreement between parents and children when the disease is more active. In this case, child’s symptoms and disease perception are more likely to be under/overestimated by parents [5, 8], without a clear direction in the difference as showed by our findings. In a previous report on JIA focusing on proxy and adolescents’ agreement, Lal et al. showed that their evaluations of pain and well-being were more likely to disagree in those with severe disease. They also observed that higher levels of agreement were most representative of a high level of well being. They did not find an explanation for this phenomenon [5].

Conversely, in an earlier report focusing on agreement for pain, general well-being, functional disability, and HRQOL in JIA patients, Shaw et al. found maximum discordance in those with only moderate disease activity [7]. April et al. explained their finding of higher levels of agreement on pain for JIA patients with more severe disease, arguing that children with more severe disease tended to have more pain and might have expressed this to their parents [11].

Also, parents might give more attention to those who have a more severe disease.

Our results show that a longer disease and the age of the patient do not affect the discordance between children and their parents, as also stated by Shaw et al. [7].

The divergence between proxy and children’s reports does not imply that only the child’s perception reflects reality. Opposing views may reflect their differing perspectives and are equally valid [12]. Parents may vary in their perception of child health concerns, and therefore health professionals should consider both children’s and parents’ perspectives. The physician has to consider this different perception and understand how it will impact on JIA management and outcome. The integration of these elements in patients care, could improve reliability of patients-parents reports on disease activity, the adherence to treatment and at the end disease management.

This study, looking not only on the global sample but comparing patient/parent dyads duo, extends the perspective of JAMAR questionnaire’s use in the clinical setting as compared to a previous publication [4].

Our study has some limitations. We used a convenience sample that had a relatively low level of disease activity, which is representative of our current clinical practice. Children and their parents were not physically separated when completing the questionnaires, which may have biased some responses.

Our study is cross-sectional and causality cannot be established. Longitudinal follow-up studies would allow a better understanding of the effective disease’s impact on health and quality of life and the impact of discordance on disease’s outcome.

## Conclusions

JAMAR is an important tool to explore different disease’s dimensions, and to give clinicians a large amount of information on divergent child and parent’s disease perceptions. The differences between patient’s and parent’s reports emphasize the importance for professionals to obtain precise information from both parents and children to improve disease management. It is essential that both patients and parents fill the JAMAR questionnaire for a complete clinical and psychosocial evaluation.

## Additional files

Additional file 1: Figure S1. a) age at visit, b) age at disease onset, c) disease duration and d) n° of actives joints in the six groups with results of ANOVA test between groups. (PDF 152 kb) Additional file 2: Table S1. Groups demographic and clinical variables. Legend: Values are expressed as means. (PDF 24 kb) Additional file 3: Table S2. Tuckey post-hoc analysis between groups for MD VAS. Legend: statistically significant differences between groups in red. (PDF 50 kb) Additional file 4: Table S3. Tuckey post-hoc analysis between groups for JADAS. Legend: statistically significant differences between groups in red. (PDF 55 kb)
