# Supplementary material for: The difference of disease perception by juvenile idiopathic arthritis patients and their parents: analysis of the JAMAR questionnaire
Source: Pediatr Rheumatol Online J. 2016 Jan 6;14:2. doi: 10.1186/s12969-015-0063-3 (PMC4702328; doi:10.1186/s12969-015-0063-3)
Supplement: Additional file 1: Figure S1. — a) age at visit, b) age at disease onset, c) disease duration and d) n° of actives joints in the six groups with results of ANOVA test between groups. (PDF 152 kb) [file 12969_2015_63_MOESM1_ESM.pdf]

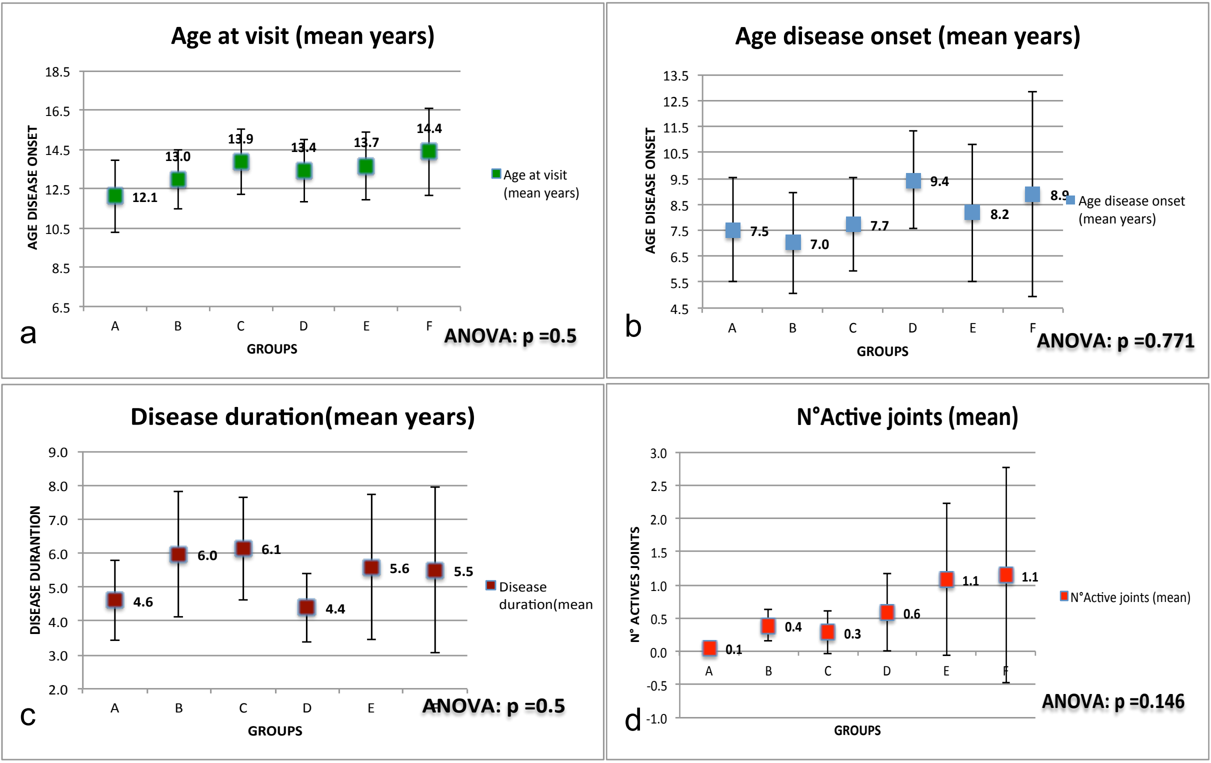

Age at visit (mean years)
AGE DISEASE ONSET
18.5
16.5
14.5
12.5
10.5
8.5
6.5
12.1
13.0
13.9
13.4
13.7
14.4
A
B
C
D
E
F
GROUPS
Age at visit (mean years)
a
ANOVA: p =0.5
Age disease onset (mean years)
AGE DISEASE ONSET
13.5
12.5
11.5
10.5
9.5
8.5
7.5
6.5
5.5
4.5
7.5
7.0
7.7
9.4
8.2
8.9
A
B
C
D
E
F
GROUPS
Age disease onset (mean years)
b
ANOVA: p =0.771
Disease duration(mean years)
DISEASE DURANTION
9.0
8.0
7.0
6.0
5.0
4.0
3.0
2.0
4.6
6.0
6.1
4.4
5.6
5.5
A
B
C
D
E
GROUPS
Disease duration(mean
c
ANOVA: p =0.5
N°Active joints (mean)
N° ACTIVES JOINTS
3.0
2.5
2.0
1.5
1.0
0.5
0.0
-0.5
-1.0
0.1
0.4
0.3
0.6
1.1
1.1
A
B
C
D
E
F
GROUPS
N°Active joints (mean)
d
ANOVA: p =0.146
