# Supplementary material for: The difference of disease perception by juvenile idiopathic arthritis patients and their parents: analysis of the JAMAR questionnaire
Source: Pediatr Rheumatol Online J. 2016 Jan 6;14:2. doi: 10.1186/s12969-015-0063-3 (PMC4702328; doi:10.1186/s12969-015-0063-3)
Supplement: Additional file 2: Table S1. — Groups demographic and clinical variables. Legend: Values are expressed as means. (PDF 24 kb) [file 12969_2015_63_MOESM2_ESM.pdf]

	Number of pairs	Age disease onset (years)	Age at visit (years)	Disease duration (years)	N°Active joints	MD global VAS	JADAS
Group A	20	7.5	12.1	4.6	0.1	0.4	1.8
Group B	23	7.0	13.0	6.0	0.4	1.4	3.7
Group C	14	7.7	13.9	6.1	0.3	1.5	6.0
Group B	24	9.4	13.4	4.4	0.6	1.9	6.2
Group E	12	8.2	13.7	5.6	1.1	3.0	8.5
Group F	7	8.9	14.4	5.5	1.1	2.4	8.0
