# Supplementary material for: The difference of disease perception by juvenile idiopathic arthritis patients and their parents: analysis of the JAMAR questionnaire
Source: Pediatr Rheumatol Online J. 2016 Jan 6;14:2. doi: 10.1186/s12969-015-0063-3 (PMC4702328; doi:10.1186/s12969-015-0063-3)
Supplement: Additional file 3: Table S2. — Tuckey post-hoc analysis between groups for MD VAS. Legend: statistically significant differences between groups in red. (PDF 50 kb) [file 12969_2015_63_MOESM3_ESM.pdf]

Dependent Variable:MD VAS							
	(I) group	(J) group	Mean Difference (I-J)	Std. Error	Sig.	95% Confidence Interval	
						Lower Bound	Upper Bound
Tukey HSD	A	B	-1.01	0.5654	,480	-2.655	0.635
		C	-1.111	0.6444	,520	-2.985	0.764
		D	-1.492	0.5599	,092	-3.121	0.137
		E	-2.617*	0.6752	**,003**	-4.581	-0.652
		F	-1.932	0.8121	,174	-4.295	0.431
	B	A	1.01	0.5654	,480	-0.635	2.655
		C	-0.101	0.6268	1,000	-1.925	1.723
		D	-0.482	0.5396	,947	-2.052	1.088
		E	-1.607	0.6585	,153	-3.523	0.309
		F	-0.922	0.7982	,857	-3.245	1.4
	C	A	1.111	0.6444	,520	-0.764	2.985
		B	0.101	0.6268	1,000	-1.723	1.925
		D	-0.381	0.6219	,990	-2.19	1.428
		E	-1.506	0.7275	,312	-3.622	0.611
		F	-0.821	0.856	,929	-3.312	1.669
	D	A	1.492	0.5599	,092	-0.137	3.121
		B	0.482	0.5396	,947	-1.088	2.052
		C	0.381	0.6219	,990	-1.428	2.19
		E	-1.125	0.6538	,522	-3.027	0.777
		F	-0.44	0.7944	,994	-2.752	1.871
	E	A	2.617*	0.6752	**,003**	0.652	4.581
		B	1.607	0.6585	,153	-0.309	3.523
		C	1.506	0.7275	,312	-0.611	3.622
		D	1.125	0.6538	,522	-0.777	3.027
		F	0.685	0.8795	,971	-1.874	3.243
	F	A	1.932	0.8121	,174	-0.431	4.295
		B	0.922	0.7982	,857	-1.4	3.245
		C	0.821	0.856	,929	-1.669	3.312
		D	0.44	0.7944	,994	-1.871	2.752
		E	-0.685	0.8795	,971	-3.243	1.874

Based on observed means.

The error term is Mean Square(Error) = 3,420.

*. The mean difference is significant at the ,05 level.
