# Supplementary material for: The difference of disease perception by juvenile idiopathic arthritis patients and their parents: analysis of the JAMAR questionnaire
Source: Pediatr Rheumatol Online J. 2016 Jan 6;14:2. doi: 10.1186/s12969-015-0063-3 (PMC4702328; doi:10.1186/s12969-015-0063-3)
Supplement: Additional file 4: Table S3. — Tuckey post-hoc analysis between groups for JADAS. Legend: statistically significant differences between groups in red. (PDF 55 kb) [file 12969_2015_63_MOESM4_ESM.pdf]

Multiple Comparisons							
Dependent Variable:JADAS							
	(I) group	(J) group	Mean Difference (I-J)	Std. Error	Sig.	95% Confidence Interval	
						Lower Bound	Upper Bound
Tukey HSD	A	B	-1.962	1.3765	,712	-5.967	2.043
		C	-4.283	1.5689	,079	-8.847	0.282
		D	-4.444*	1.3631	**,019**	-8.41	-0.478
		E	-6.748*	1.644	**,001**	-11.531	-1.965
		F	-6.240*	1.9772	**,025**	-11.992	-0.488
	B	A	1.962	1.3765	,712	-2.043	5.967
		C	-2.321	1.5262	,652	-6.761	2.119
		D	-2.482	1.3137	,415	-6.305	1.34
		E	-4.787*	1.6033	**,041**	-9.451	-0.122
		F	-4.278	1.9435	,247	-9.933	1.376
	C	A	4.283	1.5689	,079	-0.282	8.847
		B	2.321	1.5262	,652	-2.119	6.761
		D	-0.161	1.5141	1,000	-4.566	4.244
		E	-2.465	1.7712	,732	-7.618	2.688
		F	-1.957	2.0841	,935	-8.021	4.106
	D	A	4.444*	1.3631	**,019**	0.478	8.41
		B	2.482	1.3137	,415	-1.34	6.305
		C	0.161	1.5141	1,000	-4.244	4.566
		E	-2.304	1.5918	,698	-6.935	2.327
		F	-1.796	1.934	,938	-7.423	3.831
	E	A	6.748*	1.644	**,001**	1.965	11.531
		B	4.787*	1.6033	**,041**	0.122	9.451
		C	2.465	1.7712	,732	-2.688	7.618
		D	2.304	1.5918	,698	-2.327	6.935
		F	0.508	2.1413	1,000	-5.721	6.738
	F	A	6.240*	1.9772	**,025**	0.488	11.992
		B	4.278	1.9435	,247	-1.376	9.933
		C	1.957	2.0841	,935	-4.106	8.021
		D	1.796	1.934	,938	-3.831	7.423
		E	-0.508	2.1413	1,000	-6.738	5.721

Based on observed means.

The error term is Mean Square(Error) = 3,420.

*. The mean difference is significant at the ,05 level.
